# Atherogenic Index of Plasma in Non-Alcoholic Fatty Liver Disease: Systematic Review and Meta-Analysis

**DOI:** 10.3390/biomedicines10092101

**Published:** 2022-08-27

**Authors:** Abdulrahman Ismaiel, Oana Sabina Ciobanu, Mohamed Ismaiel, Daniel-Corneliu Leucuta, Stefan-Lucian Popa, Liliana David, Dilara Ensar, Nahlah Al Srouji, Dan L. Dumitrascu

**Affiliations:** 1 2nd Department of Internal Medicine, Iuliu Hatieganu University of Medicine and Pharmacy, 400006 Cluj-Napoca, Romania; 2 Faculty of Medicine, Iuliu Hatieganu University of Medicine and Pharmacy, 400006 Cluj-Napoca, Romania; 3 Department of Surgery, St Michael’s Hospital, A96 D628 Dublin, Ireland; 4 Department of Medical Informatics and Biostatistics, Iuliu Hatieganu University of Medicine and Pharmacy, 400349 Cluj-Napoca, Romania; 5 Department of Medicine, Tallaght University Hospital, D24 NR0A Dublin, Ireland

**Keywords:** non-alcoholic fatty liver disease (NAFLD), metabolic-dysfunction-associated fatty liver disease (MAFLD), hepatic steatosis, liver fibrosis, biomarkers, scores, non-invasive diagnosis

## Abstract

(1) Background: Approximately a billion people worldwide are affected by NAFLD, which places a high clinical burden and financial cost on society. Liver biopsy is the gold standard for diagnosing NAFLD, but its invasivity limits the early diagnosis of NAFLD. Hence, it is important to look for alternate techniques in detecting and diagnosing NAFLD. NAFLD is associated with atherosclerosis. The purpose of this study was to assess the effectiveness of the atherogenic index of plasma (AIP) as a non-invasive modality for predicting NAFLD. (2) Methods: A search using electronic databases PubMed, EMBASE, and Scopus was carried out to find observational studies, looking at research that had been published up until the date of 11 May 2022. The included studies’ quality, risk of bias, and internal validity were evaluated using the QUADAS-2 quality assessment tool. The key summary outcomes were the mean difference (MD) and area under the curve (AUC). (3) Results: A total of eight studies (81,178 participants) were included in our review, while 17% of the included participants had NAFLD. A sex distribution of 57.8% men and 42.2% women was observed. The AIP between NAFLD and the controls was not significant (MD 0.212 [95% CI 0.231–0.655]). A significant MD in AIP between the males and females with NAFLD was observed (MD 0.246 [95% CI 0.098–0.395]). The AIP predicted NAFLD with an AUC of 0.764 as well as in males (AUC 0.761) and females (AUC 0.733). (4) Conclusions: There was a substantial MD in the AIP between both sexes, but there was no significant difference in the AIP values between patients with NAFLD and the controls. The AIP is a reliable biomarker for the diagnosis of NAFLD since its ability to predict the development of NAFLD was comparable to that of the other biomarkers.

## 1. Introduction

Non-alcoholic fatty liver disease (NAFLD) is a common liver disease characterized by the presence of excessive fat build up within hepatocytes, in the absence of other conditions that result in hepatic steatosis and with little to no alcohol consumption [1,2]. It refers to a broad range of conditions including steatosis, non-alcoholic steatohepatitis (NASH), and cirrhosis [3]. In addition, it is a significant public health problem, prevalent in both developing and developed countries [4]. Approximately a billion people worldwide are affected by NAFLD, resulting in a substantial economic and medical burden on societies [5,6].

NAFLD diagnosis involves the exclusion of secondary causes resulting in hepatocellular fat aggregation and the presence of imagistic or histopathological evidence of hepatic steatosis [7]. Histopathological evaluation using liver biopsy is the gold standard for evaluating hepatic steatosis and diagnosing NAFLD, however, this invasive diagnostic modality comes with its limitations including high cost, sampling errors, and post procedure complications [8]. Hence, multiple studies have investigated several non-invasive modalities for NAFLD diagnosis including potential biomarkers [9,10,11,12,13].

The pathogenesis of NAFLD is complex [5] and not fully elucidated [14], involving macrovascular hepatic steatosis which disseminates across the hepatic acini with disease progression [14]. Nevertheless, the term NAFLD has been proposed to be replaced by metabolic-dysfunction-associated fatty liver disease (MAFLD) to reflect the pathogenesis more appropriately [15]. Several risk factors have been associated with the progression of fatty liver disease including insulin resistance, diabetes mellitus, hypertension, and obesity [16,17].

Moreover, patients with NAFLD often have dyslipidemia, which is atherogenic in nature and is defined by elevated serum triglyceride levels and reduced high density lipoproteins cholesterol (HDL-C) levels [18,19,20]. Hence, NAFLD patients present an increased CV risk [21,22,23]. The atherogenic index of plasma (AIP), a novel quantitative index utilized to assess lipid levels, is a strong marker of dyslipidemia [24,25]. The logarithmic ratio between triglyceride levels and HDL-C yields the AIP, which indicates the association between atherogenic and protective lipoprotein [26]. Furthermore, high AIP levels have also been linked with metabolic syndrome [27]. Several studies have revealed the high accuracy of AIP in strongly predicting the risk of several conditions such as atherosclerosis, coronary artery disease, myocardial infarction [28,29], and hyperuricemia [30].

Lately, AIP has been investigated as a potential predictive marker for the detection of NAFLD, with conflicting results. Therefore, we conducted a systematic review and meta-analysis to evaluate the AIP levels in NAFLD patients and assess its accuracy in predicting NAFLD. We also assessed the effect of sex on AIP in patients with NAFLD.

## 2. Materials and Methods

This systematic review and meta-analysis were composed as per the Preferred Reporting Items for Systematic Reviews and Meta-Analyses (PRISMA) 2020 statement [31]. The study was registered in INPLASY (International Platform of Registered Systematic Review and Meta-analysis Protocols); registration number (INPLASY202280043) [32].

### 2.1. Data Sources and Search Strategy

An electronic search of several databases including PubMed, EMBASE, and Scopus was carried out by two investigators (A.I. and O.S.C.) to find observational studies looking at AIP in NAFLD that had been published up until the date of 11 May 2022. No publication filters were used during the search.

To find the required articles from PubMed, the following search strategy was used: ((“Non-alcoholic Fatty Liver Disease”[Mesh]) OR (“Non-alcoholic Fatty Liver Disease”[All Fields]) OR (NASH) OR (“metabolic dysfunction associated fatty liver disease”) OR (“metabolic-dysfunction-associated fatty liver disease”) OR (“metabolic associated fatty liver disease”) OR (MAFLD)) AND ((“Atherogenic index of plasma”) OR (AIP)), while a similar search was performed for the EMBASE and Scopus electronic databases.

The search results’ titles and abstracts were analyzed for eligibility, and then the full text was assessed to make sure the inclusion and exclusion criteria were satisfied. The author (O.S.C.) extracted the data from the included articles, and another verified it (A.I.). The name of the author, year and country of publication, study design, total sample size, population under study, percentage of patients with NAFLD in the sample size, the technique used to diagnose NAFLD, mean age, gender ratio, body mass index (BMI), mean ± standard deviation or median of AIP of the sample, and area under the receiver operating characteristic (AUROC) curve were among the data that were extracted.

### 2.2. Eligibility Criteria

The inclusion criteria for this study comprised of the observational cohort, case-control, or cross-sectional studies assessing AIP in NAFLD; hepatic steatosis evaluated through imaging such as ultrasonography or histologically through liver biopsy; human studies without restriction to gender, race, or ethnicity; and studies published in English. The exclusion criteria were studies including patients with secondary hepatic steatosis due to other causes; conference abstracts or papers, posters, published abstracts without full articles, letters, notes, and editorials; studies including patients with the polycystic ovarian syndrome; participants under the age of 18; and interventional studies.

### 2.3. Risk of Bias Assessment in Individual Studies

The included studies’ quality, bias, and internal validity were evaluated using the QUADAS-2 quality assessment tool [33] by two investigators (O.S.C. and M.I.). In case of disagreement between the investigators, a consensus was reached through discussion. Answers for each assessment category were “yes,” “no”, or “unclear.” The QUADAS-2 assessment had no impact on the eligibility of the studies.

### 2.4. Summary Measures and Synthesis of Results

The data collection for the systematic review and the meta-analysis were analyzed in R with the Metafor package in OpenMeta[Analyst] [34,35]. The association between AIP and NAFLD was assessed using the mean difference (MD) and AUC, which examined the accuracy of AIP in predicting NAFLD. The χ2-based Q-test and I^2^ were used to assess the heterogeneity between studies. Heterogeneity was categorized using the Cochrane Handbook; I^2^ values between 0% and 40% were not important, 30% to 60% were moderate, 50% to 90% were substantial, and 75% to 100% were significant [36].

From studies that reported the median and interquartile range (IQR), the mean and standard deviation (SD) were calculated. The confidence interval and the point estimate were conducted to identify the AUC’s standard error. Statistics were combined in some studies with multiple subgroups to assess the mean and SD for the entire sample size. Moreover, subgroup analysis according to the AIP levels and AUROC in different sexes was evaluated. The Cochrane Handbook’s recommendations were followed while conducting this. For all of the meta-analyses, restricted maximum likelihood random-effects models were used. The data of the included studies were analyzed as the mean differences with a 95% confidence interval, lower bound, upper bound, standard error, and *p*-value, or as AUCs with the same set of parameters. Statistical significance was defined as a *p*-value < 0.05. An analysis was performed only when there were at least two studies that reported AIP values with the mean and SD or median and IQR, or AUC with upper and lower confidence intervals.

## 3. Results

### 3.1. General Results

**Figure 1:**
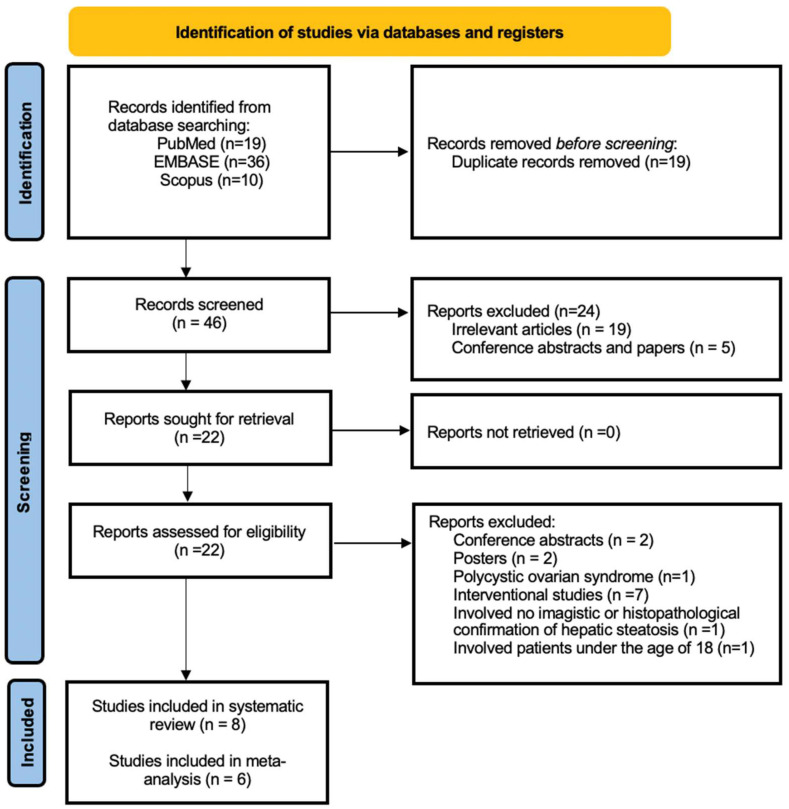
The PRISMA flow diagram of the identification, screening, and inclusion methodology of the studies.

The initial search performed retrieved a total of 65 articles: 19 articles from PubMed, 36 articles from EMBASE, and 10 articles from Scopus, as shown in Figure 1, out of which 19 articles were duplicates and were removed. Subsequently, 46 articles were screened to evaluate whether the titles and abstracts matched the inclusion and exclusion criteria. A total of 24 articles were excluded in this screening phase, where five articles were either conference abstracts or papers, while 19 articles were irrelevant studies to the subject of this review. After retrieving the remaining 22 articles, the full texts of each one was carefully reviewed to determine their eligibility. The following four causes led to the exclusion of a 14 further articles: two publications were conference abstracts [37,38]; two of the publications were posters [39,40]; one study involved patients with polycystic ovarian syndrome [41]; one study enrolled patients under the age of 18 [42]; one study involved no imagistic or histopathological confirmation of hepatic steatosis [43]; and seven publications were interventional studies [44,45,46,47,48,49,50]. Eight publications in total [4,5,51,52,53,54,55,56] were included in the systematic review, and six of these [4,5,51,52,55,56] were also included in the meta-analysis.

### 3.2. Study Characteristics

This systematic review and meta-analysis comprised 81,178 participants in total, with a sex distribution of 46,919 (57.8%) men and 34,259 (42.2%) women. A sample of 13,879 out of the total number of participants had NAFLD (17.1%). Seven studies were conducted in Asia (China, *n* = 4; Iran, *n* = 3) [4,5,51,52,53,54,55] and one in Europe (Czech Republic) [56]. Table S1 provides an overview of the key aspects of the included research.

Ultrasonography was the preferred diagnostic method in most of the included studies (*n* = 6) [4,5,52,54,55,56]. Histopathological analysis of a liver biopsy was performed in one study [51], and one study included patients who were identified using a combination of the two aforementioned techniques [53].

### 3.3. AIP and NAFLD

**Figure 2:**
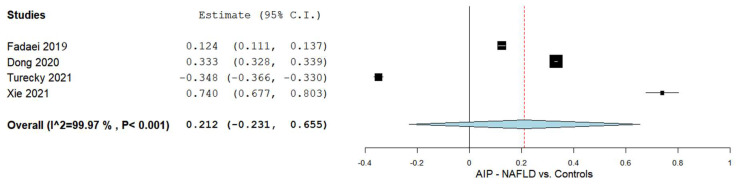
The AIP in the NAFLD patients vs. the controls. AIP—Atherogenic index of plasma; NAFLD—Non-alcoholic fatty liver disease.

The AIP levels between the NAFLD patients and controls were compared in four studies [4,5,55,56]. The results of the pooled analysis are shown in Figure 2. The results revealed an overall MD of 0.212 with a 95% CI of −0.231–0.655. Significant heterogeneity was noted as I^2^ = 99.97% with a *p*-value of < 0.001.

### 3.4. AIP and Males and Females

**Figure 3:**
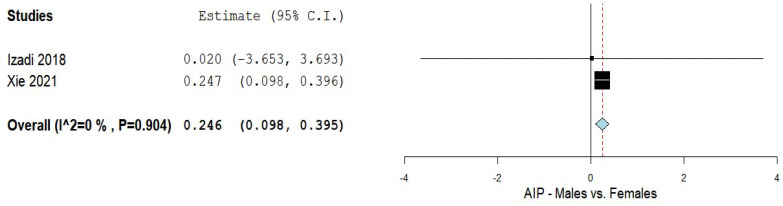
The AIP in the male vs. female NAFLD patients; AIP—Atherogenic index of plasma; NAFLD–Non-alcoholic fatty liver disease.

The AIP levels in patients with NAFLD in both genders were compared in two studies [5,51]. The results of the pooled analysis are shown in Figure 3. The analysis yielded a MD of 0.246 with a 95% CI of 0.098–0.395. A non-significant heterogeneity was noted with an I^2^ = 0% and a *p*-value = 0.904.

### 3.5. AIP in Predicting NAFLD

**Figure 4:**
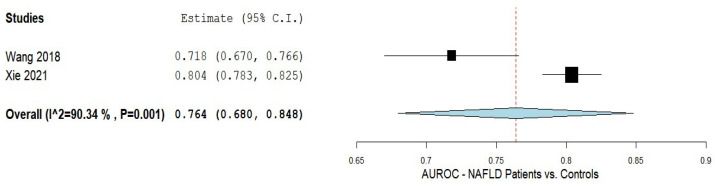
The accuracy of the AIP in predicting NAFLD. AIP—Atherogenic index of plasma; NAFLD—Non-alcoholic fatty liver disease.

The efficacy of AIP in predicting NAFLD was assessed in two studies [5,52]. The results of the pooled analysis are shown in Figure 4. An AUC of 0.764 with a 95% CI of 0.680–0.848 was noted with substantial heterogeneity of I^2^ = 90.34% and a *p*-value of < 0.001.

### 3.6. AIP in Predicting NAFLD Based on Sex

**Figure 5:**
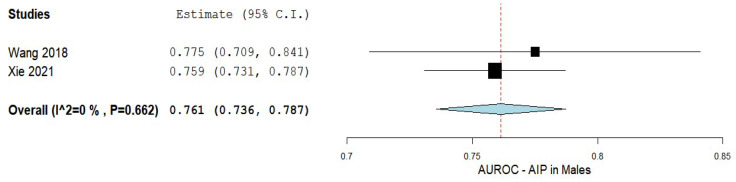
The accuracy of AIP in predicting NAFLD in men. AIP—Atherogenic index of plasma; NAFLD—Non-alcoholic fatty liver disease.

The accuracy of AIP in predicting NAFLD in patients of both male and female sexes was assessed using data from two studies. The findings of the pooled analysis for males are shown in Figure 5 [5,52]. The analysis showed an AUC of 0.761 with a 95% CI of 0.736−0.787. A non-significant heterogeneity was composed of an I^2^ = 0% and *p*-value = 0.662. 

**Figure 6:**
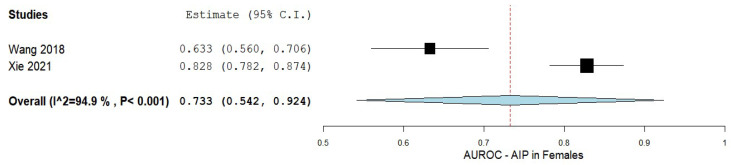
The accuracy of AIP in predicting NAFLD in females. AIP—Atherogenic index of plasma; NAFLD—Non-alcoholic fatty liver disease.

In contrast, AIP prediction in female patients with NAFLD showed an AUC of 0.733 with a 95% CI of 0.542–0.924, and a considerable heterogeneity of I^2^ = 94.9%, and a *p*-value < 0.001. Figure 6 displays a summary of the findings.

### 3.7. Bias Evaluation

As shown in Table S2, the QUADAS-2 tool was used to assess the bias risk of the included studies. There were several instances of high risk for bias in the included studies. One study out of the eight included studies used histopathology as the reference test for all of their participants [51]. Six studies used ultrasonography [4,5,52,54,55,56], and one study combined histopathology and ultrasonography [53]. Additionally, none of the studies included any details about the histological or ultrasonographic severity findings of their patients or the criteria used to evaluate the liver biopsy sample. As a result, there could be bias in the evaluation or reference test technique. Furthermore, it was not made apparent in any of the papers of how much time had passed between the reference standard and the index test. Nevertheless, it is more likely that the time was not as long as to have had an important difference between the liver situation (which does not change fast) and the AIP test. Five of the eight included studies used a case-control design, and none of the eight studies disclosed whether the patients recruited for their investigations were chosen consecutively or at random from the accessible sample.

In addition, it was not obvious whether the reference test was carried out with the knowledge of the index test; however, it is improbable that this happened. For each study, the risk of bias was assessed using the key below: NA stands for not applicable, low risk, high risk, or unclear.

## 4. Discussion

The gold standard for accurately diagnosing NAFLD is the pathological assessment of liver biopsies; however, numerous markers for NAFLD have been investigated in an endeavor to refrain from such an invasive technique. In our systematic review and meta-analysis, we assessed the AIP values in NAFLD and the accuracy of its predictability to detect NAFLD. We included eight studies with a total population of 81,178, among which six studies were included in our quantitative synthesis. We reported that the AIP levels were not significantly different in patients with NAFLD compared to the controls. However, a significant difference in the AIP levels between males and females with NAFLD was observed. The AIP predicted NAFLD (AUC 0.764) for males was AUC 0.761 and for the females it was AUC 0.733. Our results revealed that AIP possesses an acceptable predictive value for the diagnosis of NAFLD in comparison to the other investigated non-invasive biomarkers.

We reported numerous findings that require further elaboration. The NAFLD prevalence in this study was 17.1%, with a comparable sex distribution of 57.8% males and 42.2% females. We reported a significant difference in the AIP values between the males and females, with the former having higher values. Differences in the AIP levels between genders could be attributed to the discrepancies in fat storage, with the males having a greater predisposition for visceral adipose tissue build-up than the females, which supports the fact that BMI, a marker of overall adiposity, solely could be a misleading marker. Not only that, but another explanation for this sex difference could be attributed to hormonal and lifestyle factors [57].

The average BMI of the included studies was 28 kg/m^2^. Lonardo et al. revealed that a high BMI is an independent predictor of fatty liver in both genders [58]. Visceral adiposity, in subjects with normal and high BMI, is a risk factor for several conditions including metabolic syndrome and cardiovascular disease [58]. Numerous studies have described the association between visceral adiposity and fatty liver [59]. NAFLD could affect obese and non-obese individuals. When it occurs in the latter, it is called lean NAFLD, which is characterized as having a BMI of <25 kg/m^2^ [60].

Moreover, most of the participants were largely from two main countries: China and Iran. Hence, the generalizability of the obtained results cannot be performed on races and ethnicities that have not been studied yet. There is no clear consensus on the AIP cut-off for NAFLD prediction between different races. Hence, different cut-offs were observed among various studies of diverse ethnicities. Giannini et al. revealed discrepancies in the TG/HDL-C ratio between different ethnicities [61].

We reported no significant difference in AIP between patients with NAFLD and the controls. This finding could be attributable to the imbalance between the number of NAFLD patients and controls in most of the included studies, the former being more than that of the controls. The majority of the studies did not provide sufficient information on the characteristics of the controls limiting our interpretation. Even though no apparent significance was observed between the NAFLD patients and controls, our results revealed that AIP possesses an adequate predictive value for the diagnosis of NAFLD (AUC 0.764), which was comparable to the other markers. Ismaiel et al. revealed that the visceral adiposity index (VAI) had an AUC of 0.767 for NAFLD [11]. Additionally, FLI, HIS, NAFLD-LFS, and the SteatoTest had an AUROC of 0.84, 0.81, 0.86–0.87, and 0.79–0.80, respectively [62]. This accuracy implies that AIP could be utilized as it is a non-invasive, easily calculated, and non-costly marker that could aid in the early diagnosis of NAFLD, reducing the cost burden and associated complications of NAFLD.

The present guidelines consider the use of liver biopsy as the gold standard for the accurate diagnosis of NAFLD [63]. Nevertheless, seven of the eight studies used ultrasonography instead. As such, the exact prevalence of NAFLD within the included studies may have been underestimated. Additionally, due to the observational nature of the included studies, causality between AIP and NAFLD could not be confirmed or inferred. Several non-invasive markers for the identification of advanced fibrosis such as NAFLD fibrosis score, and FIB-4 index have been reported [60]. Vilar-Gomez et al. also showed that the FIB-4 index and NFS were effective markers for distinguishing liver fibrosis stages [64]. The FIB-4 index is calculated based on age, AST, ALT, and platelet count. In our study, we could not investigate the association between AIP and liver fibrosis as a limited number of the included studies reported the degree of liver fibrosis of the included patients, not allowing us to conduct a subgroup analysis.

It is important to note that the term NAFLD has been renamed MAFLD. This new term is much more generalized than NAFLD, and its diagnostic criteria are composed of hepatic steatosis with the presence of overweight/obesity, T2DM, or metabolic dysregulation [65]. Our study only analyzed its results and findings solely through the criteria of NAFLD rather than MAFLD.

BMI and liver enzymes solely are weak and inaccurate screening markers for NAFLD. Thus, the use of a marker such as AIP is promising. Not only that, but it has been described as a potential tool for the screening of MAFLD [27]. The AIP is also useful in reflecting the interaction between protective and atherogenic lipoproteins. It can be merely calculated from the blood lipid levels, particularly triglycerides and HDL-C. It is an indirect indicator of visceral adiposity, and several studies have described the link between obesity, dyslipidemia, and adipose tissue dysfunction [66,67].

Dysregulation of the hepatic lipid metabolic pathways can result in lipid accumulation, leading to oxidative stress, hepatocyte injury, and the development and progression of NAFLD [68]. It has also been shown that high AIP values (i.e., high TG/HDL-C ratio) could indicate insulin resistance [52], which is another factor closely associated with NAFLD. However, it has been revealed that this was not the case in certain ethnicities such as African-Americans [69].

Insulin resistance, which is significantly associated with visceral fat, is the metabolic syndrome’s cornerstone [70]. When cells in the muscles and fat as well as hepatocytes do not present proper insulin effects, this condition is known as insulin resistance. Therefore, compared to the general population, patients with insulin resistance present difficulty in metabolizing blood glucose, which causes the pancreas to produce more insulin to facilitate the entry of glucose into the cells. Additionally, insulin resistance appears to play a key role in the pathogenesis of NAFLD by determining a rise in hepatic lipogenesis and a lack of the inhibition of adipose tissue lipolysis, resulting in a rise in the flow of fatty acids into the liver [71]. A major contributor to the development of dyslipidemia associated with NAFLD and a main cause of morbidity and mortality in NAFLD patients, premature cardiovascular disease, is insulin resistance [72]. Interestingly, a recent study has demonstrated a link between the prevention of type 2 diabetes and the prolonged clearance of hepatitis C virus by direct-acting antivirals through reducing insulin resistance [73].

Combining several non-invasive tests such as the FIB-4 index and liver stiffness measurement by vibration-controlled transient elastography for the diagnosis of NAFLD and advanced fibrosis has been shown to enhance the specificity and sensitivity [74,75]. Furthermore, numerous studies have investigated the value of non-invasive markers such as NFS as an indicator of mortality in NAFLD patients [76]. Hence, it would be beneficial to assess the combination of AIP and other non-invasive markers in the diagnosis of NAFLD and its use as a prognostic marker.

We had several limitations in our systematic review and meta-analysis. This study included observational design studies, and hence causality between the AIP and NAFLD could not be concluded. Most of the included studies used ultrasonography for the diagnosis of NAFLD, which could have resulted in the underestimation of the NAFLD prevalence. There is a scarce number of studies that have examined the AIP in NAFLD patients contributing to the limited literature. Hence, our systematic review and meta-analysis included a limited number of studies. NAFLD and insulin resistance are strongly associated; hence it is valuable to analyze AIP in NAFLD patients with and without diabetes. However, very few of the included studies stated the diabetes profile of the recruited individuals, thus limiting our ability to stratify the AIP results based on the diabetes profile. Additionally, there is a lack of consensus on the cut-off of AIP for detecting NAFLD. Half of the included studies had a high risk of bias involving patient selection, by and large due to the lack of reporting of consecutive/random sampling utilization and the application of a case-control design, both of which could affect the generalizability of the results and presence of selection bias. None of the included studies reported the methods in which the biopsy or ultrasonography was performed, nor the experience of the operators, which may have influenced the overall prevalence of NAFLD in the included studies and the accuracy of the reference test. The sample’s diversity of racial and ethnic backgrounds was constrained by the fact that most of the included studies were from Asia, bar one study from Europe, which limits the generalizability of the results; hence it would be worthwhile investigating the AIP on a broader scale. It should be noted that some of our subgroup analyses comprised a few studies, which could have impacted the power of the results. However, it is important to note that the key results from our study were based on an adequate number of subjects.

Moreover, our systematic review and meta-analysis has numerous strengths. The prevalence of NAFLD and its associated conditions and complications, coupled with its significant medical burden, entails the recognition of a non-invasive modality for the screening of patients who are more likely to have NAFLD. We revealed results with respect to the accuracy of AIP in predicting NAFLD, which is comparable to published data from other non-invasive indices and markers. To the best of our knowledge, our review non-biasedly focused on a topic that has not been fully investigated to date. Additionally, we used a thorough search strategy encompassing numerous medical databases, which enabled us to systematically investigate the association between the AIP and NAFLD. Six out of the eight included studies used the same tests across all of the subjects, which makes the results more reliable.

## 5. Conclusions

There was a significant difference in the AIP between male and female patients with NAFLD; however, no apparent significant mean difference between NAFLD and the controls was observed. When compared to the currently recommended scores and other non-invasive markers, it was concluded that the AIP’s accuracy in predicting NAFLD was acceptable. Triglycerides, HDL, and cholesterol are regularly tested markers; therefore, calculating the AIP in patients would be easily achievable. Patients with high AIP can undergo further investigations to rule out metabolic disorders such as NAFLD. A limited number of studies have evaluated the accuracy of AIP as a non-invasive marker for predicting the development of NAFLD; hence, future research is warranted.

## Data Availability

Not applicable.

## Supplementary Materials

The following supporting information can be downloaded at: https://www.mdpi.com/article/10.3390/biomedicines10092101/s1. Table S1: A summary of the main characteristics of the included studies; Table S2: The QUADAS-2 tool used to evaluate the methodological quality of the first four included studies.
