# Supplementary material for: Atherogenic Index of Plasma in Non-Alcoholic Fatty Liver Disease: Systematic Review and Meta-Analysis
Source: Biomedicines. 2022 Aug 27;10(9):2101. doi: 10.3390/biomedicines10092101 (PMC9495578; doi:10.3390/biomedicines10092101)
Supplement: Supplementary file 1 [file biomedicines-10-02101-s001.zip › 2. Supplementary Tables.pdf]

Table S1. Summary of the main characteristics of the included studies

First Author / Country	Year	Study Design	Study Characteristics	
Izadi et al. / Iran	2018	Cross-sectional study	**Total Subjects:**	83
			**Population:**	Patients with confirmed diagnosis of NAFLD from the university hospital in Jahrom, Iran
			**NAFLD:**	100%
			**Mean age (years):**	36.71 ± 7.21
			**Gender (males):**	42 (50%)
			**BMI (kg/m2):**	29.41 ± 4.18
			**NAFLD diagnosis:**	Histological
			**AIP - Mean ± SD / Median (IQR):**	**Male:** 0.716 ± 0.16<br>**Female:** 0.696 ± 12
			**AIP – AUC:**	-
Fadaei et al. / Iran	2019	Case control study	**Total Subjects:**	82
			**Population:**	Patients were recruited from Shariati Hospital (Tehran, Iran)
			**NAFLD:**	49 (59.8%)

			**Mean age (years):**	**Control**: 50<br>**NAFLD**: 51
			**Gender (males):**	41
			**BMI:**	**Control**: 25.0 ± 0.6<br>**NAFLD**: 28.0 ± 0.4
			**NAFLD diagnosis:**	Ultrasonography
			**AIP - Mean ± SD / Median (IQR):**	**Control**: 0.374 ± 0.032<br>**NAFLD:** 0.498 ± 0.026
			**AIP – AUC:**	-
Wang et al. / China	2018	Observational study	**Total Subjects:**	538
			**Population:**	Data was collected at local health stations by trained medical staff in the Shandong Province, China
			**NAFLD:**	392 (73%)
			**Mean age (years):**	**Low AIP level:** 42.18 ± 11.59 **Intermediate AIP level:** 42.2 ± 11.2<br>**High AIP level:** 41.7 ± 11.5
			**Gender (males):**	281 (52.23%)
			**BMI:**	**Low AIP level:** 30.48 ± 2.16 **Intermediate AIP level**: 30.63 ± 2.03<br>**High AIP level:** 30.97 ± 2.21

			**NAFLD diagnosis:**	Ultrasonography
			**AIP - Mean ± SD / Median (IQR):**	**Low AIP level:** −0.11 ± 0.15 **Intermediate AIP level:** 0.16 ± 0.03<br>**High AIP level:** 0.48 ± 0.28
			**AIP – AUC:**	**Total:** 0.718 (0.670–0.766)<br>**Male:** 0.775 (0.709-0.841)<br>**Female:** 0.633 (0.561-0.706)
Izadi et al. / Iran	2019	Cross-sectional study	**Total Subjects:**	83
			**Population:**	Patients from Jahrom University of Medical Science
			**NAFLD:**	100%
			**Mean age (years):**	36.71 ± 7.21
			**Gender (males):**	42 (50.6%)
			**BMI:**	29.41 ± 4.18
			**NAFLD diagnosis:**	Ultrasonography, histopathological
			**AIP - Mean ± SD / Median (IQR):**	0.697 ± 0.153
			**AIP – AUC:**	-
Liu et al. / China	2020	Observational Study	**Total Subjects:**	250

			**Population:**	Patients with T2DM who visited and were followed up at the Endocrinology Department of Affiliated Hospital 2 of Nantong University and 50 healthy controls from the Department of Physical Examination Center between Apr 2017 and Oct 2018
			**NAFLD:**	89 (35.6%)
			**Mean age (years):**	**Controls:** 35.3 ± 10.9<br>**Total T2DM:** 52.4 ± 10.7
			**Gender (males):**	**Controls:** 19.6%<br>**T2DM Total:** 60.5%
			**BMI:**	**Controls:** 22.5 ± 2.7<br>**T2DM Total:** 25.8±3.6
			**NAFLD diagnosis:**	Hepatic steatosis on ultrasound
			**AIP - Mean ± SD / Median (IQR):**	**Controls:** -0.13±0.30<br>**Total T2DM:** 0.23±0.25
			**AIP – AUC:**	-
Dong et al. / China	2020	Cross-Sectional Study	**Total Subjects:**	78,304
			**Population:**	Chinese population from First Affiliated Hospital of Wenzhou Medical University between Jan 2010 - Dec 2014

			**NAFLD:**	**Development Group:** NAFLD: 8904 (6.16%) **Validation Group:** NAFLD: 3689 (6.35%)
			**Mean age (years):**	**Development Group:** NAFLD: 49.4±13.5 Non-NAFLD: 43.7 ± 15.6 **Validation Group:** NAFLD: 49.7 ± 13.8 Non-NAFLD: 43.6 ± 15.6
			**Gender (males):**	45,096 (57.6%)
			**BMI:**	**Development Group:** NAFLD: 23.4±1.2 Non-NAFLD: 21.3±2.1 **Validation Group:** NAFLD: 23.4±1.2 Non-NAFLD: 21.3±2.0
			**NAFLD diagnosis:**	Ultrasonography
			**AIP - Mean ± SD / Median (IQR):**	**Development Group:** NAFLD: 0.2 (0.0,0.4) Non-NAFLD:−0.1 (− 0.3,0.0) **Validation Group:** NAFLD: 0.2 (0.0,0.4) Non-NAFLD:−0.1 (− 0.3,0.0)
			**AIP – AUC:**	**Development:** 0.803 (0.798–0.808) **Validation:** 0.802 90.795–0.810)
Turecký et al. / Czech Republic	2021	Observational Study	**Total Subjects:**	90
			**Population:**	**Control:** 26 people **NAFLD:** 64 patients
			**NAFLD:**	64 (71.1%)
			**Mean age (years):**	**Control:** 35.6

				**NAFLD:** 55.2
			**Gender (males)**:	**Control:** 43 (42.3%)<br>**NAFLD:** 32 (50%)
			**BMI**:	-
			**NAFLD diagnosis**:	Ultrasound and positive fatty liver index
			**AIP - Mean ± SD / Median (IQR):**	**Control:** -0.191 ± 0.04<br>**NAFLD:** 0.157 ±0.04
			**AIP – AUC:**	-
Xie et al. / China	2021	Cross-Sectional Study	Total Subjects:	1748
			**Population:**	Recruited in physical examination center of Suzhou.
			**NAFLD:**	526 (30.09%)
			**Mean age (years):**	**Fatty liver (FL) (+)** = 46 (38, 57),<br>**FL (-)** = 43 (36, 58)
			**Gender (males)**:	**FL (+)** = 464 (26.5%),<br>**FL (-)** = 689 (39.42%)
			**BMI**:	**FL (+)** = 26.5 (24.6, 28.5),<br>**FL (-)** = 22.7 (20.5, 24.5)
			**NAFLD diagnosis**:	Ultrasonography

			**AIP - Mean ± SD / Median (IQR):**	**FL (+)** = 0.59 (0.13, 0.98),<br>**FL (-)** = -0.18 (-0.56, 0.22)
			**AIP - Mean ± SD / Median (IQR) M:F:**	**Male:** **FL (+):** 0.63 (0.16, 0.99) **FL (-):** −0.03 (−0.037, 0.039)<br>**Female:** **FL (+):** 0.33 (− 0.01, 0.72) **FL (-):** − 0.42 (− 0.76, 0)
			**AIP – AUC:**	**Total:** 0.804 (0.783-0.825)<br>**Males:** 0.759 (0.731, 0.787)<br>**Females**: 0.828 (0.782, 0.874)

Table S2. QUADAS-2 tool used to evaluate the methodological quality of first four included studies

**Criteria**	**Izadi 2018**	**Fadaei 2019**	**Wang 2018**	**Izadi 2020**
**A. Risk of bias**				
Was a consecutive or random sample of patients enrolled?	Unclear	Unclear	Unclear	Unclear
Was a case-control design avoided?	Yes	No	Yes	Yes
Did the study avoid inappropriate exclusions?	Yes	Yes	Yes	Yes
Risk of bias assessment	Low risk	High risk	Low risk	Low risk
**B. Applicability**	Low risk	Low risk	Low risk	Low risk
**A. Risk of bias**				
Were the index test results interpreted without knowledge of the results of the reference standard?	Yes	Yes	Yes	Yes
If a threshold was used, was it pre-specified?	NA	NA	NA	NA
Risk of bias assessment	Low risk	Low risk	Low risk	Low risk
**B. Applicability**	Low risk	Low risk	Low risk	Low risk
**A. Risk of bias**				
Is the reference standard likely to correctly classify the target condition?	Yes	No	No	Yes
Were the reference standard results interpreted without knowledge of the results of the index test?	Yes	Yes	Yes	Yes
Risk of bias assessment	Low risk	Low risk	Low risk	Low risk
**B. Applicability**	Low risk	Low risk	Low risk	Low risk
**A. Risk of bias**				
Was there an appropriate interval between index test(s) and reference standard?	Unclear	Unclear	Unclear	Unclear
Did all patients receive a reference standard?	Yes	Yes	Yes	Yes
Did patients receive the same reference standard?	Yes	Yes	Yes	Yes
Were all patients included in the analysis?	Yes	Yes	Yes	Yes
Risk of bias assessment	Low risk	Low risk	Low risk	Low risk

Table S2 (Cont'd). QUADAS-2 tool used to evaluate the methodological quality of last four included studies

**Criteria**	**Liu 2020**	**Dong 2020**	**Turecký 2021**	**Xie 2021**
**A. Risk of bias**				
Was a consecutive or random sample of patients enrolled?	Unclear	Unclear	Unclear	Unclear
Was a case-control design avoided?	No	No	No	No
Did the study avoid inappropriate exclusions?	Yes	Yes	Yes	Yes
Risk of bias assessment	High risk	High risk	High risk	High risk
**B. Applicability**	Low risk	Low risk	Low risk	Low risk
**A. Risk of bias**				
Were the index test results interpreted without knowledge of the results of the reference standard?	Yes	Yes	Yes	Yes
If a threshold was used, was it pre-specified?	NA	NA	NA	NA
Risk of bias assessment	Low risk	Low risk	Low risk	Low risk
**B. Applicability**	Low risk	Low risk	Low risk	Low risk
**A. Risk of bias**				
Is the reference standard likely to correctly classify the target condition?	No	No	No	No
Were the reference standard results interpreted without knowledge of the results of the index test?	Yes	Yes	Yes	Yes
Risk of bias assessment	Low risk	Low risk	Low risk	Low risk
**B. Applicability**	Low risk	Low risk	Low risk	Low risk
**A. Risk of bias**				
Was there an appropriate interval between index test(s) and reference standard?	Unclear	Unclear	Unclear	Unclear
Did all patients receive a reference standard?	Yes	Yes	Yes	Yes
Did patients receive the same reference standard?	Yes	Yes	Yes	Yes
Were all patients included in the analysis?	Yes	Yes	Yes	Yes
Risk of bias assessment	Low risk	Low risk	Low risk	Low risk
